# Conflict and community: mental health in the Arab world

**DOI:** 10.1192/bji.2020.59

**Published:** 2021-02

**Authors:** David Skuse

**Affiliations:** Professor of Behavioural and Brain Sciences, Division of Population, Policy and Practice, UCL Great Ormond Street Institute of Child Health, London, UK. Email: d.skuse@ucl.ac.uk

**Keywords:** Arab, institutionalisation, in-patient, refugees, stigma

## Abstract

This month's issue of *BJPsych International* focuses on the Middle East, with papers on psychiatric care in conflict zones, the persistence of institutionalisation in Arab countries, service delivery in Iraq, improved media attitudes towards mental illness in Qatar and integration of mental health services into primary care in that country.

Our theme this month concerns countries throughout the Arab world where there are major changes taking place in the recognition of mental disorders and delivery of services. As our authors Joseph El-Khoury, Andres Barkil-Oteo and Lynn Adam acknowledge, the Middle East has been an area of conflict for decades. Currently, 8 out of 20 Arab League of Nations states are involved in armed struggles.^1^ The impact on the mental health of their populations is a matter of international concern because waves of refugees are putting pressure on services in those other countries, within and outside the region, that are willing to accept them. The authors discuss how the conventional definition of post-traumatic stress disorder may be an inappropriate descriptor of the associated emotional and behavioural challenges faced by their psychiatric infrastructures. Specific and valuable recommendations are made about how to achieve better outcomes for the mental health of a traumatised populace, in both the short and long term.

In a related article, Joelle Abi-Rached considers a controversial topic, the continuing use of large-scale hospitals to contain the mentally ill in some countries of the region.^2^ Whereas a revolution in community treatment took root in many Western societies 50 years ago, followed by the discontinuation of institutional care, she presents evidence (and an accompanying video) that psychiatric in-patient services are expanding – even in high-income Arab countries. Her discussion about why institutionalisation persists is controversial and provocative.

Then Aws Sadik gives us a valuable snapshot of Iraqi psychiatry as it is practised today, 17 years after a coalition of Western countries visited ‘shock and awe’ on the citizens of that country, destroying its infrastructure and destabilising the Middle East for decades to come.^3^ Interestingly, an Iraqi Mental Health Act was passed in 2005, but its provisions are not widely implemented. Although there remain many challenges to delivering services to the mentally ill, enduring stigma being one of them, there is evidence that new models of primary care are being developed. Unfortunately, sectarianism and corruption persist, and prevailing attitudes toward girls and women constitute an additional impediment to progress.

We end this series of articles with good news about developments in Qatar, one of the wealthiest countries in the region. Khalid Elzamzamy and colleagues have reviewed the attitudes of the Qatar daily press to mental health issues, comparing their reporting with that on physical health.^4^ There are encouraging signs that stigmatising those with mental disorders is rare nowadays, and negative articles complain instead about the limitations of services to meet demand. The authors conclude that the media's portrayal of mental health in Qatar is more positive than in many other countries worldwide. Finally, Ovais Wadoo and colleagues discuss the history of mental health services in Qatar, which were established in 1971.^5^ A National Mental Health Strategy was published in 2014, following which there has been a trend to provide mental health services in collaboration with family physicians, emphasising the value of community-based specialist care. Qatar is leading the way with this enlightened vision. Mental healthcare is being integrated with the primary healthcare system and a training infrastructure to support it is being built too. The authors conclude that Qatar thus provides a model for other countries in the Arabian Gulf region to follow.
